# Effects of Caffeine Mouth Rinse on Cognitive Performance and Psychological Responses in Trained Individuals: A Systematic Review

**DOI:** 10.3390/nu18213695

**Published:** 2026-11-07

**Authors:** Chieh-Ju Lu, Chi-Lun Tsai, Kuo-Pin Wang

**Affiliations:** 1 Master Program of Sport Facility Management and Health Promotion, National Taiwan University, Taipei 10617, Taiwan; r12h42014@g.ntu.edu.tw; 2 Department of Sport Psychology, Faculty of Sport Science, Universität Leipzig, 04109 Leipzig, Germany; chi-lun.tsai@studserv.uni-leipzig.de; 3 NTU Plus Academy, National Taiwan University, Taipei 106319, Taiwan; 4 Center for Cognitive Interaction Technology (CITEC), Bielefeld University, 33619 Bielefeld, Germany

**Keywords:** caffeine mouth rinse, cognitive performance, psychological responses, physically trained individuals, sports nutrition

## Abstract

**Background:** Caffeine mouth rinse (Caf-MR) has been investigated as a non-ingestive strategy that may influence cognitive performance and psychological responses. However, evidence in trained individuals remains limited, and psychological responses have not been comprehensively synthesized. This systematic review evaluated the effects of Caf-MR on cognitive performance and psychological responses in trained individuals. **Methods:** Six electronic databases were searched through 24 September 2026 for randomized controlled studies comparing Caf-MR with a non-caffeinated placebo or control. Reference lists of relevant reviews and eligible studies were also screened. Owing to heterogeneity in protocols and outcomes, findings were synthesized qualitatively. **Results:** Ten studies were included; five assessed cognitive outcomes and six psychological responses, with one study contributing to both domains. Cognitive findings were heterogeneous, with improvements observed in selected reaction-time measures and Stroop interference time, as well as fewer errors during a sport-specific cognitive–motor task, while other reaction-time and accuracy outcomes showed no clear benefit. Among psychological studies, only one reported a Caf-MR-specific increase in felt arousal, with no consistent effects on mood, motivation, mental effort, mental fatigue, or enjoyment. Methodological quality ranged from fair to good, and all studies had some concerns regarding overall risk of bias. **Conclusions:** Overall, despite improvements in selected cognitive outcomes, current evidence remains insufficient to establish consistent cognitive or psychological benefits of Caf-MR in trained individuals. Future research should prioritize assessing blinding effectiveness, comparing Caf-MR protocols under standardized experimental conditions, and using comparable outcome measures to clarify potential differences across tasks and contexts.

## 1. Introduction

In sport and exercise contexts, maintaining cognitive performance and adaptive psychological states is important for trained individuals [1,2]. As competition or exercise progresses, accumulating physical and mental demands may impair cognitive functioning and alter psychological states, potentially compromising performance [3,4]. For example, prolonged endurance exercise may alter arousal (i.e., the subjective level of activation, ranging from low states such as drowsiness or relaxation to high states such as alertness or excitement [5]) and increase mental fatigue [6,7], whereas sports requiring rapid reactions and decision-making may be affected by declines in processing speed under cumulative mental demands [8,9]. These changes highlight the need for practical strategies that can be implemented before or during sport and exercise to support cognitive functioning and psychological states. Nutritional interventions are particularly relevant because they can be administered acutely and integrated relatively easily into training and competition routines, and may influence psychological responses and cognitive processing [10,11]. Therefore, identifying nutritional strategies that can support cognitive performance and psychological states during training and competition may have practical value for trained individuals.

Caffeine is one of the most widely investigated ergogenic aids in sports science [12,13]. Previous studies have shown that caffeine ingestion may improve endurance performance, muscular power, and sport-specific performance [12,14,15]. In addition to its effects on physical performance, caffeine has been suggested to improve cognitive processes, such as reaction time, attentional control, and vigilance, and to modulate psychological states, including arousal, motivation, and mood [10,11,16]. Although low-to-moderate doses of ingested caffeine are generally well tolerated, higher doses may increase the risk of gastrointestinal discomfort, headache, anxiety, or other undesirable psychophysiological symptoms, particularly in susceptible individuals [17]. These limitations have encouraged interest in alternative strategies that may provide acute stimulation while minimizing the need for gastrointestinal absorption.

Caffeine mouth rinse (Caf-MR) has been proposed as a non-ingestive strategy that may act through oral sensory stimulation [18,19]. By stimulating oral sensory receptors, potentially including bitter taste receptors, Caf-MR may influence neural pathways associated with information processing and reward [20]. Related research using carbohydrate mouth rinsing has also examined mood and perceived arousal in trained triathletes, although clear psychological benefits attributable specifically to mouth rinsing were not established [21]. However, whether the proposed oral sensory mechanisms of Caf-MR translate into consistent improvements in cognitive performance remains unclear. A previous systematic review suggested that Caf-MR may be beneficial for cognitive performance [22], whereas more recent meta-analytic evidence indicated less consistent overall effects, with speed-based outcomes appearing more responsive than accuracy-based outcomes after sensitivity analysis [23]. These discrepancies may partly reflect differences in cognitive outcome measures, participant characteristics, Caf-MR protocols, and experimental conditions across the included studies.

However, previous evidence syntheses have generally included heterogeneous participant populations, limiting conclusions that can be drawn specifically for trained individuals. This distinction is particularly relevant in sport settings, where cognitive and psychological demands, such as rapid decision-making, sustained attention, and fatigue-related challenges, are frequently encountered during training and competition. Moreover, previous evidence suggests that training status may influence responses to caffeine ingestion [13,24], although whether similar differences occur following Caf-MR remains unclear. Therefore, the present review specifically synthesizes cognitive and psychological outcomes following Caf-MR in individuals with structured sport- or exercise-training backgrounds, addressing a population-specific evidence gap not resolved by previous reviews.

Alongside these cognitive investigations, increasing attention has also been given to the effects of Caf-MR on psychological responses, such as arousal, motivation, affective states, mental fatigue, and enjoyment. Although these outcomes have been examined in experimental studies, they have not been comprehensively synthesized in previous systematic reviews. Previous reviews and meta-analyses have instead primarily focused on exercise performance and cognitive outcomes [22,23,25,26]. Therefore, the broader psychological responses to Caf-MR remain insufficiently characterized.

To address these gaps, this systematic review aimed to examine the effects of Caf-MR on cognitive performance and psychological responses in trained individuals. By specifically synthesizing evidence on cognitive and psychological outcomes among individuals engaged in structured sport or exercise training, this review aimed to provide a more focused and comprehensive understanding of Caf-MR responses and to clarify whether its potential effects extend beyond cognitive performance to broader psychological responses relevant to training and competition. Given the proposed oral sensory mechanisms and the mixed findings reported in previous studies, it was hypothesized that Caf-MR would improve selected aspects of cognitive performance and influence psychological responses in this population.

## 2. Material and Methods

### 2.1. General Guidelines

This systematic review was conducted in accordance with the PRISMA 2020 guidelines [27]. A PRISMA flow diagram was used to summarize the study selection process. The review was retrospectively registered with the International Platform of Registered Systematic Review and Meta-analysis Protocols (INPLASY; registration number: INPLASY202690016) after completion of the review procedures and prior to manuscript submission. The retrospective registration was undertaken to provide a transparent and publicly accessible record of the review methodology. Ethics approval and informed consent were not required because this review used data from previously published studies.

### 2.2. Database Searches and Identification of Eligible Studies

Two authors (L-CJ and T-CL) independently conducted electronic database searches in PubMed, Scopus, Web of Science, ScienceDirect, Embase, and the Cochrane Library to identify relevant original studies. In addition, the reference lists of relevant reviews and eligible studies were manually screened to identify additional records. The search strategy was developed using predefined terms related to caffeine mouth rinsing and cognitive or psychological outcomes, combined using Boolean operators. The caffeine mouth-rinse terms included (caffeine OR caffeinated OR coffee) AND (“mouth rinse” OR “mouth rinsing” OR “oral rinse” OR “oral rinsing” OR mouthwash). For cognitive outcomes, these terms were combined with (cognit* OR attention* OR “reaction time” OR vigilance OR memory OR “executive function” OR “inhibitory control” OR psychomotor). For psychological outcomes, they were combined with (psycho* OR mood OR affect* OR arousal OR motivation* OR pleasure OR “mental fatigue” OR feeling*). Search syntax and field restrictions were adapted as necessary for each database. The detailed database-specific search strategies are provided in the Supplementary Material (Supplementary Table S1). The literature search and study selection were conducted independently by two reviewers (L-CJ and T-CL). Disagreements were resolved through discussion, with W-KP consulted when consensus could not be reached. All databases were last searched on 24 September 2026.

### 2.3. Inclusion and Exclusion Criteria

Eligibility criteria were defined according to the PICO framework: (1) Population: trained individuals; (2) Intervention: caffeine mouth rinse; (3) Comparison: a non-caffeinated placebo or control condition; and (4) Outcomes: cognitive performance and/or psychological responses. Specifically, studies were included if they met all of the following criteria: (1) They used a randomized controlled design. (2) They involved physically trained individuals, defined as those regularly participating in structured sport- or exercise-specific training. Training status was assessed based on information reported in the original studies, using the training-related criteria for the trained category in previously published classification frameworks or the criteria above [28,29]. When detailed quantitative training data were unavailable, documented participation in organized competitive sports, together with evidence of regular structured training, was considered sufficient to support classification as physically trained. Studies lacking sufficient information to support this classification were excluded. (3) They investigated caffeine mouth rinse as the intervention. (4) They included a non-caffeinated placebo or control condition. (5) They assessed cognitive performance and/or psychological responses. (6) They were published as peer-reviewed articles in English and reported original experimental data.

Studies were excluded if they met any of the following criteria: (1) participants were untrained or were described only as physically or recreationally active without sufficient information to establish regular structured sport- or exercise-specific training; (2) caffeine was administered only by ingestion, without a Caf-MR intervention; (3) they were reviews, abstracts, or non-original reports; (4) they did not assess cognitive performance and/or psychological responses; (5) they investigated caffeine mouth rinsing combined with carbohydrate in the same solution, without a separate caffeine-only mouth-rinse condition.

### 2.4. Data Extraction

Data were extracted independently by two reviewers (L-CJ and T-CL). Disagreements were resolved through discussion, with W-KP consulted when consensus could not be reached. Extracted information included participant characteristics, training status, habitual caffeine intake, study design, Caf-MR protocols, control conditions, dietary control, blinding procedures, outcome measures, and main findings relevant to the eligible outcomes. Eligible outcomes were categorized as cognitive or psychological responses. Studies reporting both cognitive and psychological outcomes were included in both categories. Information that was not reported was recorded as not available.

Considerable heterogeneity in intervention protocols, outcome measures, and experimental conditions was observed across the ten included studies. Caf-MR protocols varied in rinse duration (5–30 s), solution volume (25–250 mL), caffeine concentration or dose, and administration frequency (single versus repeated rinsing). Only five studies assessed cognitive outcomes and six assessed psychological outcomes, using different tasks and measurement instruments. Cognitive assessments included reaction-time measures, Stroop and flanker tasks, and sport-specific cognitive–motor assessments, whereas psychological outcomes encompassed distinct constructs, including arousal, mood, motivation, mental effort, mental fatigue, and enjoyment. Although three studies assessed felt arousal using the same scale, differences in rinsing protocols and experimental conditions limited direct comparisons. Variations in assessment timing further reduced comparability across studies. Given these differences and the limited number of studies assessing sufficiently comparable outcomes, quantitative pooling was considered inappropriate. Findings were therefore synthesized qualitatively and separately for cognitive performance and psychological responses.

### 2.5. Methodological Quality and Risk-of-Bias Assessment

Methodological quality was assessed using a modified version of the Physiotherapy Evidence Database (PEDro) scale [30]. Consistent with previous research [12], one additional item was included to assess whether each study formally evaluated the effectiveness of participant blinding. Since Item 1 (eligibility criteria) was not scored, the total modified PEDro score ranged from 0 to 11, with studies classified as excellent (10–11), good (7–9), fair (5–6), or poor (<5). Risk of bias was assessed using the Cochrane Risk of Bias 2 (RoB 2) tool for randomized crossover trials [31]. The assessed domains included the randomization process, risk of bias arising from period and carryover effects, deviations from intended interventions, missing outcome data, measurement of the outcome, and selection of the reported result. Each domain and the overall risk of bias were judged as “low risk,” “some concerns,” or “high risk” according to the RoB 2 guidance. Both the PEDro and RoB 2 assessments were conducted independently by two reviewers (L-CJ and T-CL). Disagreements were resolved through discussion, with a third reviewer (W-KP) consulted when consensus could not be reached.

## 3. Results

### 3.1. Study Identification and Selection

**Figure 1:**
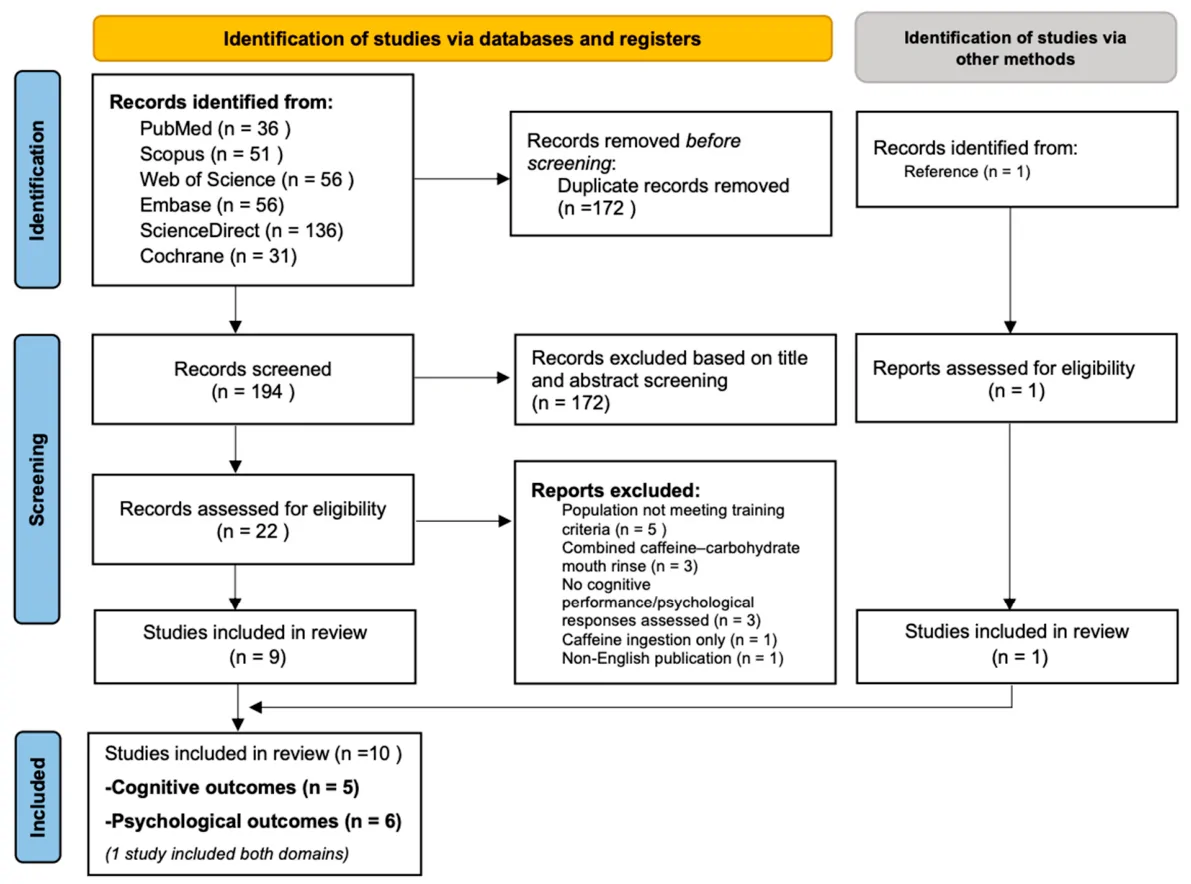
PRISMA 2020 flowchart for the current systematic review.

The PRISMA flow diagram summarizing the study selection process is presented in Figure 1. After duplicate records were removed, the remaining records were screened based on their titles and abstracts. Records that did not meet the eligibility criteria with respect to caffeine mouth rinsing, trained participants, or cognitive or psychological outcomes were excluded. Following full-text assessment, a total of ten studies met the eligibility criteria and were included in this review. Among the included studies, five examined cognitive performance outcomes [32,33,34,35,36], whereas six examined psychological responses [18,32,37,38,39,40]. One study contributed to both outcome categories [32]. The detailed characteristics of the included studies are summarized in Table 1 and Table 2.

### 3.2. Characteristics of Studies Examining Cognitive Performance

Across the five studies examining cognitive outcomes (Table 1), the total sample comprised 130 participants, including 98 males and 32 females. All study populations met the review’s definition of trained individuals and included resistance-trained team-sport athletes [32], trained volleyball or football athletes [33], female table-tennis players [34], trained karate athletes [35], and well-trained volleyball players [36]. Habitual caffeine intake was reported in three studies. Karayigit et al. [32] reported a very low habitual caffeine intake of approximately 20 mg/day, Virdinli et al. [33] reported an intake of 163 mg/day, and Amor et al. [36] reported an intake of 1.14 ± 0.11 mg/kg/day. Habitual caffeine intake was not reported in the remaining two studies [34,35].

Caf-MR protocols varied considerably across studies. Rinse duration ranged from 5 to 15 s. Two studies used a 10 s rinse [33,35], one used repeated 10 s rinses [32], one used 5 s rinses [34], and one used a 10–15 s rinse [36]. A 25 mL mouth-rinse volume was used in four studies [32,33,34,35], whereas Amor et al. [36] used 250 mL. Caffeine was administered at concentrations ranging from 1.2% to 2.4% in three studies [32,33,35]. Pirmohammadi et al. [34] used espresso containing approximately 60 mg of caffeine per 25 mL, whereas Amor et al. [36] prescribed caffeine relative to body mass at 3 mg/kg. Regarding rinse frequency, three studies used a single rinse [33,35,36], Pirmohammadi et al. [34] administered three 5 s rinses at 10, 5, and 0 min before testing, and Karayigit et al. [32] administered eight 10 s rinses at 1 min intervals.

The timing and type of cognitive assessments also differed across studies. Four studies assessed cognitive outcomes before exercise or in a non-exercise testing setting [32,33,34,36], whereas Şahin et al. [35] assessed reaction time following a fatigue-inducing repeated-sprint and kicking protocol. Cognitive outcomes included simple and choice reaction time and Stroop performance [36], hand and foot visual reaction time [33], hand and kick reaction time [35], reaction time and response accuracy during a modified flanker task [32], and performance on a sport-specific color-response cognitive test [34].

### 3.3. Characteristics of Studies Examining Psychological Responses

Across the six studies examining psychological responses (Table 2), the total sample comprised 133 participants, including 95 males and 38 females. All study populations met the review’s definition of trained individuals and included recreationally resistance-trained males [18], recreationally trained male cyclists [37], resistance-trained team-sport athletes [32], male university rugby union players [39], well-trained male youth soccer players [38], and amateur female volleyball players [40]. Habitual caffeine intake was reported in three studies. Karayigit et al. [32] reported a very low habitual caffeine intake of approximately 20 mg/day, Tallis et al. [39] reported an intake of 188 ± 88 mg/day, and Yüce et al. [40] included participants consuming <200 mg/day. Habitual caffeine intake was not reported in the remaining three studies [18,37,38].

Caf-MR protocols varied across studies. Five studies used a 10 s rinse protocol [18,32,37,38,40], whereas Tallis et al. [39] used a 30 s rinse. A 25 mL mouth-rinse volume was used in five studies [18,32,37,38,40], whereas Tallis et al. [39] used 50 mL. Among studies using fixed caffeine concentrations, concentrations ranged from 0.16% to 2%, whereas Tallis et al. [39] prescribed caffeine relative to body mass at 3 mg/kg. Three studies used a single rinse [18,38,39], whereas Boat et al. [37], Karayigit et al. [32], and Yüce et al. [40] used repeated-rinse protocols administered before or during the experimental task.

Psychological outcomes and assessment timing varied across studies. Felt arousal was assessed using the Felt Arousal Scale (FAS) [41] before and after rinsing in two studies [18,32] and before and after treatment administration in Tallis et al. [39]. Tallis et al. [39] additionally assessed readiness to invest physical (RIPE) and mental (RIME) effort using visual analog scales (VASs), consistent with previous research [42]. Boat et al. [37] assessed motivation and task importance repeatedly during a 10 km cycling trial using visual analog scales (VASs). Oliveira et al. [38] assessed enjoyment using the Exercise Enjoyment Scale (EES) [43], mental fatigue using a VAS, and mood states before and after exercise. Yüce et al. [40] assessed enjoyment using the Exercise Activity Enjoyment Scale (EAES) [43,44], mental effort using the Rating Scale of Mental Effort (RSME) [45], and mood states using the Brunel Mood Scale (BRUMS) [46,47] during or following the small-sided game protocol.

**Table 1:** Characteristics and cognitive outcomes of included studies examining the effects of caffeine mouth rinse.

Study	Study Design	Population/ Training Status	Age (Years)	Cognitive Measure/ Timing	Caf-MR Protocol	Dietary Situation/ Testing Time	Control	Main Findings
Karayigit et al. [32]	Double-blind	27 resistance-trained team-sport athletes; ≥1 year; ≥3 resistance-training sessions/week	M: 24.0 ± 3.0; F: 21.0 ± 1.0	Modified flanker task: RT + accuracy; pre-/post-rinse	2% CAF, 500 mg/25 mL; 8 × 10 s, once/min over 8 min before post-rinse cognitive test	Overnight fast; 07:00–08:00	PLA: water + sucralose	↔ RT and response accuracy
Virdinli et al. [33]	Double-blind	45 trained male volleyball/football athletes; 9 ± 3 years; 14 ± 3 h/week	18.0 ± 3.0	Hand + foot visual RT; immediately post-rinse	1.2%, 1.8%, 2.4% CAF; 25 mL; 1 × 10 s, immediately pre-RT	≥2 h after breakfast; 11:00–13:00	PLA: water rinse; Control: no rinse	↓ Hand RT (1.2%, 1.8%, 2.4%) ↓ Foot RT (1.2%, 2.4% vs. PLA) ↔ Foot RT (1.8% vs. PLA)
Pirmohammadi et al. [34]	Double-blind	18 female table-tennis players; ~3 years; national league	20.5 ± 3.1	Sport-specific color-response cognitive test; after 3 pre-test rinses	Espresso (~60 mg CAF/25 mL); 3 × 5 s at −10, −5, and 0 min pre-test	Standardized breakfast 2 h before; 10:00–11:00	PLA: 5 g starch capsule	↓ Cognitive-test errors
Şahin et al. [35]	Double-blind	16 trained male karate athletes; 7.8 years; 8.4 h/week	21.6 ± 3.4	Kick RT + hand visual RT; assessed after repeated-sprint and kicking protocol	1.2% CAF, 300 mg/25 mL; 1 × 10 s, pre-exercise	≥3 h after last meal; N/A	PLA: water + non-caloric sweetener	↔ Kick RT and hand RT
Amor et al. [36]	Double-blind	24 well-trained male volleyball players; 7.0 ± 1.0 years; ≥4 sessions/week	16.9 ± 0.7	SRT, CRT + Stroop; post-rinse	3 mg/kg CAF in 250 mL; 1 × 10–15 s, immediately pre-battery	Last meal ≥3 h before; 08:00, 12:00, 18:00	PLA: cellulose capsule + water rinse with quinine and flavoring	↓ Stroop interference time (12:00 only) ↔ SRT, CRT, and Stroop errors

Effects refer to Caf-MR compared with placebo/control. Age data are presented as mean ± standard deviation (SD) in years. ↓, statistically significant decrease; ↔, no significant effect. Caf-MR, Caffeine Mouth Rinse; CAF, Caffeine; PLA, Placebo; SRT, Simple Reaction Time; CRT, Choice Reaction Time; RT, Reaction Time; N/A, Not Available or Not Applicable.

**Table 2:** Characteristics and psychological outcomes of included studies examining the effects of caffeine mouth rinse.

Study	Study Design	Population/ Training Status	Age (Years)	Psychological Measure/ Timing	Caf-MR Protocol	Dietary Situation/ Testing Time	Control	Main Findings
Clarke et al. [18]	Double-blind	15 recreationally resistance-trained males ≥6 months; 3 ± 1 sessions/week	21.0 ± 2.0	FAS; pre- and post-rinse	1.2% CAF, 300 mg/25 mL; 1 × 10 s, pre-exercise	Same diet across trials; 09:00–12:00	PLA: water + 200 mg sucralose; Control: no rinse	↔ Felt arousal
Boat et al. [37]	Double-blind	15 endurance recreationally trained male cyclists; 8 ± 3 h/week; cycling-event training	22.4 ± 2.6	Motivation + task importance (VAS); at 1, 3, 5, 7, 9, and 10 km	0.16% CAF, 40 mg/25 mL; 5 × 10 s, pre + every 2 km	4 h postprandial; N/A	PLA: taste-, texture-, and color-matched solution	↔ Motivation and task importance
Karayigit et al. [32]	Double-blind	27 resistance-trained team-sport athletes; ≥1 year; ≥3 resistance-training sessions/week	M: 24.0 ± 3.0; F: 21.0 ± 1.0	FAS; pre-rinse and after serial rinsing	2% CAF, 500 mg/25 mL; 8 × 10 s, once/min over 8 min before post-rinse assessment	Overnight fast; 07:00–08:00	PLA: water + sucralose	↑ Felt arousal
Tallis et al. [39]	Double-blind	27 male university-standard rugby union players; regular season; experimental sessions replaced regular strength and conditioning sessions	20.0 ± 2.0	RIPE, RIME and FAS; pre- and post-administration	3 mg/kg CAF/50 mL; 1 × 30 s, 1 min pre-exercise	Same diet across trials; N/A	PLA: taste-matched solution	↔ RIPE, RIME, and FAS
Oliveira et al. [38]	Double-blind	25 well-trained male youth soccer players; ≥3 years; ≥4 sessions/week + 1 match/week	16.6 ± 0.5	EES + mental-fatigue VAS: post-LSPT; mood states: pre/post	1.2% CAF, 300 mg/25 mL; 1 × 10 s, pre-LSPT	2 h fast; testing 15:00–17:00	Control: water rinse	↔ Enjoyment, mental fatigue, and mood states
Yüce et al. [40]	Single-blind	24 amateur female volleyball players; ≥3 years; ≥4 sessions/week	17.3 ± 2.4	EAES + RSME: baseline/during/post-SSG; BRUMS: pre/post	0.8% CAF, 200 mg/25 mL; 10 s rinse before each set (INT) or every 5 min (CONT)	N/A	PLA: placebo solution	↔ Enjoyment, mental effort, and mood states

Effects refer to Caf-MR compared with placebo/control. Age data are presented as mean ± standard deviation (SD) in years. ↑, statistically significant increase; ↔, no significant effect. Caf-MR, Caffeine Mouth Rinse; PLA, Placebo; FAS, Felt Arousal Scale; RIPE, Readiness to Invest Physical Effort; RIME, Readiness to Invest Mental Effort; EES, Exercise Enjoyment Scale; EAES, Exercise Activity Enjoyment Scale; VAS, Visual Analog Scale; RSME, Rating Scale of Mental Effort; BRUMS, Brunel Mood Scale; LSPT, Loughborough Soccer Passing Test; SSG, Small-Sided Game; INT, Intermittent; CONT, Continuous; N/A, Not Available or Not Applicable.

### 3.4. Risk of Bias and Methodological Quality

**Figure 2:**
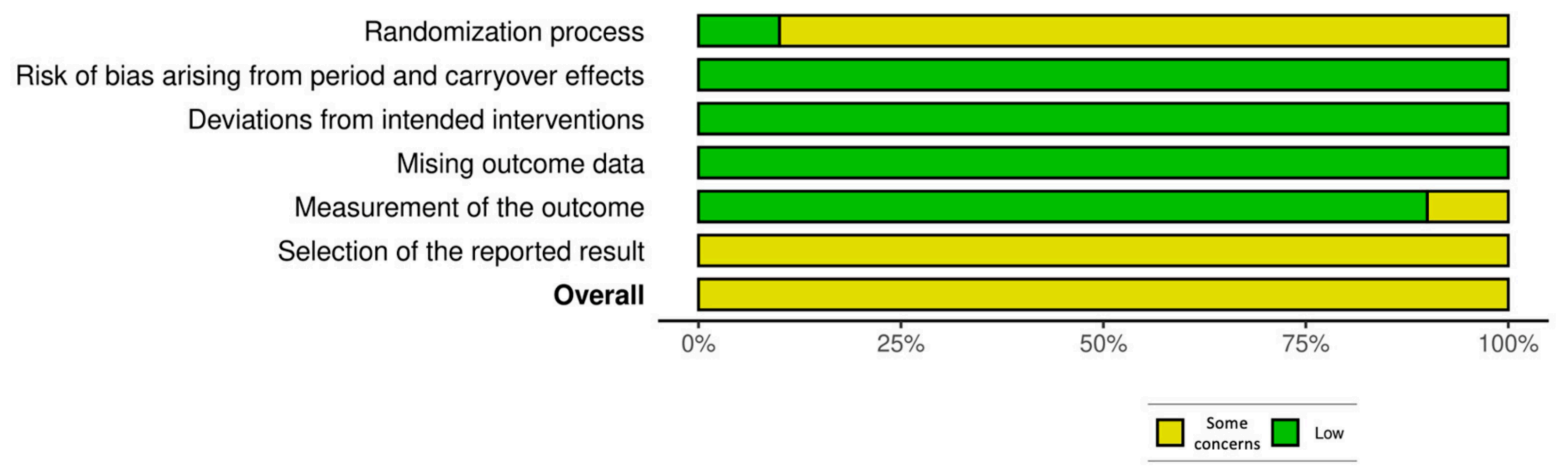
Summary of risk-of-bias assessments across the included studies using the Cochrane RoB 2 tool for crossover trials.

With respect to the overall risk of bias, no study was judged as having a low overall risk of bias. All ten included studies [18,32,33,34,35,36,37,38,39,40] were rated as having some concerns, and none were rated as having a high risk of bias (Figure 2).

In the domain-level assessment, nine studies [18,32,33,34,35,37,38,39,40] were rated as having some concerns regarding the randomization process, primarily because details regarding sequence generation or allocation concealment were insufficiently reported, whereas one study [36] was rated as low risk. Bias due to deviations from intended interventions and bias due to missing outcome data were rated as low risk across all ten studies [18,32,33,34,35,36,37,38,39,40]. For bias in measurement of the outcome, nine studies [18,32,33,34,35,36,37,38,39] were rated as low risk, whereas one study [40] was rated as having some concerns. Bias in selection of the reported result was rated as having some concerns across all ten studies [18,32,33,34,35,36,37,38,39,40], largely because study protocols or prespecified analysis plans were generally unavailable. Bias arising from period and carryover effects was rated as low risk across all ten studies [18,32,33,34,35,36,37,38,39,40], based on the reported study procedures and available information. The details of the risk-of-bias assessment are summarized in Table 3.

In addition, methodological quality was assessed using the modified PEDro scale. Four studies [33,36,37,39] scored 7–8 points and were classified as having good methodological quality, whereas six studies [18,32,34,35,38,40] scored 6 points and were classified as having fair methodological quality. The modified PEDro scores ranged from 6 to 8, with a mean score of 6.5, indicating fair-to-good methodological quality across the included studies. The detailed PEDro assessment is presented in Table 4.

## 4. Discussion

### 4.1. Cognitive Outcomes

Overall, the five studies assessing cognitive outcomes showed heterogeneous responses to Caf-MR, with effects varying according to the cognitive task and outcome assessed. Improvements were observed in selected reaction-time, interference-time, and response-accuracy measures [33,34,36], whereas other reaction-time and accuracy outcomes showed no clear benefit [32,35,36]. Virdinli et al. [33] reported faster hand reaction times at all concentrations and faster foot reaction times at selected concentrations compared with placebo. Amor et al. [36] found that Caf-MR shortened Stroop interference time at 12:00, although no significant improvements were observed in simple reaction time, choice reaction time, or Stroop interference errors. Pirmohammadi et al. [34] reported fewer errors during a sport-specific color-response cognitive task. In contrast, Karayigit et al. [32] observed no significant improvement in reaction time or response accuracy in a modified flanker task, while Şahin et al. [35] did not demonstrate a clear beneficial effect of Caf-MR on hand or kick reaction time compared with placebo. Taken together, these findings suggest that Caf-MR may benefit certain aspects of cognitive performance in trained individuals, but the observed effects varied across cognitive tasks and outcome measures.

Furthermore, the present findings partly differ from those of the existing meta-analysis [23]. After outlier removal, the meta-analysis found significant effects for speed-based cognitive outcomes, whereas effects on accuracy remained inconsistent, indicating that speed-based outcomes may be more responsive to Caf-MR. Although two studies reported improvements in response speed [33,36], Pirmohammadi et al. [34] also observed fewer errors during a sport-specific color-response task. However, this finding was based on a single study and does not establish that sport-specific tasks are more sensitive to Caf-MR than conventional cognitive assessments. One possible explanation for these differences relates to variations in task characteristics, including cognitive demands, response modalities, and outcome measures. For example, the included studies assessed visual reaction speed [33], interference control using Stroop and flanker tasks [32,36], and perceptual–motor integration through a sport-specific cognitive–motor task [34]. These tasks differ in their cognitive requirements and performance measures, which may partly contribute to the variability in observed responses to Caf-MR. Nevertheless, the limited evidence and differences in experimental conditions prevent firm conclusions regarding the influence of task characteristics. Future studies should therefore incorporate both conventional computerized cognitive tasks and sport-specific cognitive–motor assessments within standardized experimental designs to better characterize potential task-related differences in cognitive responses to Caf-MR.

The cognitive benefits observed in some studies may partly reflect the rapid engagement of central pathways involved in attention and reward processing following brief oral exposure to caffeine. Supporting this possibility, De Pauw et al. [20] demonstrated that Caf-MR increased activation within the dorsolateral prefrontal cortex and orbitofrontal cortex, regions associated with attentional control and reward processing. However, direct neurophysiological evidence regarding Caf-MR in trained individuals remains scarce. Examining neural responses specifically in trained populations is particularly important, as previous evidence suggests that sport training is associated with distinct patterns of neural processing and cortical activity [48,49,50]. Accordingly, future studies could combine electroencephalography (EEG) with cognitive assessments to characterize neural responses to Caf-MR and their relationship with cognitive performance in trained individuals.

### 4.2. Psychological Outcomes

Overall, the available evidence does not indicate a consistent Caf-MR-specific effect on psychological responses in trained individuals. Among the six included studies, only Karayigit et al. [32] reported a significant Caf-MR-specific change, showing increased felt arousal following repeated rinsing. Clarke et al. [18] observed an increase in felt arousal following rinsing, but this response did not differ significantly between experimental conditions, while Tallis et al. [39] did not demonstrate a clear Caf-MR-specific effect on felt arousal or readiness to invest physical or mental effort. Similarly, Boat et al. [37] reported no significant changes in motivation or task importance during a 10 km cycling trial. More recent studies also found no clear Caf-MR-specific effects on enjoyment, mental fatigue, mental effort, or mood states in youth soccer and volleyball players [38,40].

The observed increase in felt arousal suggests that Caf-MR may alter subjective arousal under certain conditions [32]. However, this finding was not consistently reproduced across the other studies assessing felt arousal, and no significant Caf-MR-specific effects were observed for the other psychological outcomes examined. Karayigit et al. [32] also acknowledged that the underlying mechanism remained unclear because brain activity and neurotransmitter concentrations were not assessed. Further replication is therefore needed to determine whether Caf-MR consistently affects subjective arousal.

Taken together, the inconsistent findings across cognitive and psychological outcomes may also be considered in relation to the route of caffeine administration. Amor et al. [36] reported more consistent improvements in selected cognitive outcomes following caffeine ingestion than mouth rinsing, whereas Tallis et al. [39] found no consistent advantage of ingestion for the psychological outcomes assessed. While Caf-MR may influence neural activity through oral sensory stimulation [20], ingested caffeine can exert central pharmacological effects through adenosine receptor antagonism. Animal studies have implicated adenosine A2A receptor signaling in caffeine-related effects on mood and memory [51,52], as well as age-related memory dysfunction [53]. Further animal evidence supports the involvement of neuronal A2A receptors and striatal mechanisms in caffeine-related effects on physical performance [54,55]. Collectively, these animal findings highlight the important role of caffeine’s pharmacological actions within the brain in mediating its behavioral and performance-related effects. However, these findings from animal studies cannot be directly extrapolated to Caf-MR in humans, and whether differences in the mechanisms associated with caffeine ingestion and mouth rinsing contribute to the inconsistent cognitive and psychological responses to Caf-MR remains unclear.

### 4.3. Methodological and Contextual Factors

#### 4.3.1. Rinse Protocol

Across the included studies, Caf-MR protocols varied considerably in rinse duration, frequency, caffeine concentration or dose, and solution volume. For cognitive outcomes, rinse duration ranged from 5 to 15 s, with beneficial and null findings observed across both single and repeated rinsing protocols. Cognitive benefits were reported following three 5 s rinses before cognitive testing in Pirmohammadi et al. [34], a single 10 s rinse in Virdinli et al. [33], and a single 10–15 s rinse in Amor et al. [36], whereas Şahin et al. [35] reported no clear benefit following a single 10 s rinse and Karayigit et al. [32] reported no improvement following eight serial 10 s rinses over 8 min. Taken together, these findings indicate that differences in rinse duration or frequency alone do not readily explain the mixed cognitive responses to Caf-MR. Whether repeated oral exposure provides additional cognitive benefit remains unclear because rinse frequency has not been directly compared under otherwise standardized conditions. Caffeine concentration or dose also does not clearly explain the mixed cognitive findings. Benefits and null findings were observed across substantially different dosing approaches. However, Virdinli et al. [33], the only included study to directly compare multiple caffeine concentrations, reported significantly faster hand and foot reaction times with 2.4% Caf-MR than with either 1.2% or 1.8% Caf-MR. Notably, the 1.2% concentration also significantly improved both hand and foot reaction times compared with placebo. This within-study pattern suggests that caffeine concentration may influence reaction-time responses, although further direct comparisons are needed before a dose–response relationship can be established.

For psychological outcomes, one potentially relevant pattern emerged for felt arousal. Karayigit et al. [32] observed increased felt arousal following eight serial 10 s rinses, whereas Clarke et al. [18] and Tallis et al. [39] did not observe Caf-MR-specific changes in felt arousal following single-rinse protocols. The increase in felt arousal observed by Karayigit et al. [32] following repeated rinsing is broadly consistent with previous evidence suggesting that rinse frequency may be associated with exercise-performance responses to Caf-MR [22,23]. However, within the included psychological studies, this pattern was observed only for felt arousal. Other studies using repeated rinsing did not report significant changes in motivation or task importance [37], or in enjoyment, mental effort, or mood [40]. Thus, whether rinse frequency influences psychological responses to Caf-MR remains unclear. Future studies directly comparing single and repeated rinsing protocols are needed to clarify this possibility.

#### 4.3.2. Experimental Context and Assessment Conditions

Differences in contextual and baseline conditions should also be considered when interpreting responses to Caf-MR. For cognitive outcomes, pre-test nutritional status showed a tentative but inconsistent pattern. Pirmohammadi et al. [34] provided a standardized breakfast 2 h before testing, while Virdinli et al. [33] tested participants at least 2 h after breakfast; both studies reported improvements in selected cognitive outcomes. In contrast, Karayigit et al. [32], which used an overnight fast, did not observe a cognitive benefit of Caf-MR. However, Şahin et al. [35] also reported no clear cognitive benefit despite testing participants at least 3 h after their last meal, suggesting that meal timing alone is unlikely to explain the mixed cognitive findings. Future cognitive studies should therefore standardize and clearly report pre-test nutritional status and meal timing.

Time of day may represent another contextual factor, although the available evidence remains limited. Among the included studies, only Amor et al. [36] directly compared multiple testing times within the same participants and reported an improvement in Stroop interference time following Caf-MR at 12:00, whereas no corresponding benefit was observed at 08:00 or 18:00. Other studies reporting cognitive benefits were conducted between 10:00 and 11:00 [34] and between 11:00 and 13:00 [33], whereas Karayigit et al. [32] did not observe a cognitive benefit when testing between 07:00 and 08:00. However, these other studies did not directly compare different testing times, and cross-study differences in participant characteristics, including age, cognitive tasks, Caf-MR protocols, and experimental conditions limit inferences regarding a time-of-day effect. Notably, Amor et al. [36] included adolescent athletes. Therefore, the current evidence is insufficient to establish a time-of-day effect. Further studies directly comparing different testing times under standardized experimental conditions are needed to clarify this possibility.

For psychological outcomes, assessment timing and pre-treatment baseline conditions may represent important contextual considerations. The included studies assessed psychological responses at different time points, including before Caf-MR administration [18,32,39,40], after Caf-MR administration and before exercise or task onset [18,32,39], and during or after exercise or task performance [37,38,40]. For example, Karayigit et al. [32] assessed felt arousal immediately before and after serial mouth rinsing, whereas Oliveira et al. [38] assessed enjoyment and mental fatigue only after completion of the exercise task. Yüce et al. [40] included psychological assessments at baseline, during exercise, and after exercise. Baseline assessment practices also differed, with some studies reporting pre-treatment measurements for selected psychological outcomes [18,32,39,40], whereas others did not obtain corresponding baseline values for all outcomes. Notably, Tallis et al. [39] reported that readiness to invest physical effort was already higher in the caffeine-rinse than placebo-rinse condition before treatment, indicating that the between-condition difference preceded Caf-MR administration. These differences may influence whether acute psychological responses are detected, particularly because psychological changes following acute exercise may be subtle and baseline states such as mood and mental fatigue may vary by time of day [56]. Future studies should therefore predefine and standardize psychological assessment time points, obtain corresponding pre-treatment measurements where appropriate, verify baseline comparability across conditions, and maintain consistent testing times across experimental visits.

### 4.4. Methodological Quality and Risk of Bias

Methodological quality assessed using the modified PEDro scale ranged from fair to good, indicating generally adequate methodological quality across the ten included studies. However, most studies did not clearly report whether allocation sequences were adequately concealed before condition assignment. Although insufficient reporting does not necessarily indicate inadequate concealment, the possibility that treatment sequences could have been anticipated raises concerns about selection bias and reduces confidence in the robustness of the available evidence. Consistent with the modified PEDro [12,23] approach, an additional item was used to assess whether the effectiveness of participant blinding had been formally evaluated. Among the included studies, only Virdinli et al. [33] formally evaluated blinding effectiveness. Participants were able to distinguish the caffeine mouth-rinse conditions from placebo, indicating that blinding was not fully effective. Karayigit et al. [32] explicitly acknowledged that blinding effectiveness was not assessed by asking participants to identify the solutions, while Amor et al. [36] noted that residual taste cues and participants’ beliefs regarding condition allocation may have influenced cognitive outcomes. Together, these observations suggest that expectancy effects cannot be fully excluded. If participants could distinguish caffeine-containing solutions from placebo, their expectations might have influenced subjective psychological responses, such as arousal, mood, motivation, and mental effort, as well as cognitive task performance. Such expectancy-related influences may complicate the attribution of observed effects to Caf-MR itself. However, because blinding effectiveness was rarely assessed, the extent to which these factors influenced the reported findings remains uncertain. These considerations highlight the importance of selecting comparator conditions that account for sensory characteristics and formally assessing and reporting blinding effectiveness rather than relying solely on labels such as “single-blind” or “double-blind.”

The RoB 2 assessment showed that all included studies had some concerns regarding the overall risk of bias, mainly in the randomization process and selection of the reported result. Randomization procedures were often insufficiently described, and preregistered protocols or prespecified analysis plans were generally unavailable. In contrast, deviations from intended interventions, missing outcome data, and period or carryover effects were generally judged to be at low risk. Overall, these concerns limit confidence in the reported cognitive and psychological effects of Caf-MR and highlight the need for clearer reporting and preregistration in future studies.

### 4.5. Future Directions

Building on the present synthesis, several priorities should guide future research in trained individuals. First, Caf-MR protocol variables should be examined using purpose-designed trials that directly manipulate caffeine concentration and oral exposure while controlling rinse duration, frequency, solution volume, and sensory characteristics. At present, differences in these factors are largely distributed across separate studies, making it difficult to determine their independent effects. Direct comparisons of caffeine concentrations and single versus repeated rinsing under otherwise standardized conditions are therefore needed to clarify whether caffeine concentration or oral exposure pattern influences cognitive or psychological responses.

Second, the experimental context in which Caf-MR is used requires more systematic investigation. Pre-exercise nutritional status and meal timing should be standardized, the potential influence of time of day should be further investigated, and habitual caffeine intake should be assessed and clearly reported. Future crossover trials should also directly compare fed and fasted conditions within the same participants. Such studies would help determine whether the effectiveness of Caf-MR depends on the nutritional state or testing context in which it is administered and would improve its practical application within sport nutrition strategies.

Third, cognitive assessment should be designed to distinguish different components of performance and better reflect sport-specific demands. Reaction time and response accuracy should be reported separately, as the current evidence does not establish whether Caf-MR preferentially affects either domain. Conventional computerized tasks should also be complemented, where appropriate, by sport-specific cognitive–motor assessments to determine whether laboratory effects translate to perceptual and decision-making demands relevant to sport performance.

Fourth, cognitive and psychological responses should be assessed concurrently and at predefined time points. Psychological outcomes remain understudied, and the available evidence does not indicate a consistent Caf-MR-specific effect beyond an isolated increase in felt arousal. Future studies should assess constructs such as arousal, motivation, mental effort, mental fatigue, and mood before Caf-MR administration and repeatedly during the experimental protocol. Concurrent cognitive assessment would help determine whether cognitive responses are accompanied by changes in subjective psychological states and whether these responses emerge immediately after rinsing or only under exercise- or fatigue-related conditions.

Fifth, blinding integrity and expectancy should be treated as explicit methodological considerations. Only one included study formally evaluated blinding effectiveness, and participants in that study were able to distinguish caffeine mouth-rinse conditions from placebo. Future trials should therefore carefully select comparator conditions that balance sensory matching with effective blinding, formally assess participants’ beliefs regarding condition allocation, and report blinding effectiveness. This is particularly important for subjective psychological outcomes and for studies comparing caffeine concentrations, where differences in bitterness may increase the likelihood of condition identification.

Finally, future studies should expand the evidence base on cognitive and psychological responses to Caf-MR in trained individuals, as most existing research in this population has focused primarily on exercise performance. Future studies should also clearly report training characteristics, including training experience, frequency, volume, and competitive level, to facilitate more meaningful comparisons as the evidence base grows. When possible, neurophysiological measures such as EEG may also be incorporated alongside cognitive assessments [57,58] and psychological and psychophysiological measures [59,60,61,62] to characterize the central responses associated with Caf-MR.

### 4.6. Strengths and Limitations

This review has several strengths. It appears to be the first systematic review to jointly synthesize cognitive performance and psychological responses to Caf-MR specifically in trained individuals, thereby extending the existing literature beyond its predominant focus on exercise performance. Its focus on trained individuals also improves the applicability of the findings to this specific population. In addition, cognitive and psychological outcomes were examined separately, allowing task-specific cognitive responses and distinct psychological constructs to be considered rather than combined into broad outcome categories. Methodological quality and risk of bias were also evaluated using both the modified PEDro scale and the RoB 2 tool for crossover trials. The additional assessment of blinding effectiveness was particularly relevant given the potential for participants to identify caffeine-containing solutions and the possible influence of expectancy on cognitive and psychological responses.

Several limitations should also be acknowledged. First, the evidence base remains relatively small, with only ten eligible studies and even fewer studies assessing individual cognitive or psychological outcomes. Considerable heterogeneity was present across Caf-MR protocols, cognitive tasks, psychological measures, exercise conditions, participant characteristics, and assessment timing, limiting direct comparisons across studies and precluding firm conclusions regarding optimal Caf-MR protocols or the contexts in which cognitive or psychological effects may occur. In particular, differences in participant age may confound indirect comparisons of cognitive outcomes across studies conducted at different times of day. Although the review specifically focused on trained individuals, the current evidence base was not sufficiently large to examine whether responses differ across sport types or competitive backgrounds. Habitual caffeine intake was also inconsistently reported across studies, limiting assessment of its potential influence on cognitive or psychological responses to Caf-MR. In addition, relatively few studies assessed cognitive and psychological outcomes concurrently, limiting conclusions regarding whether cognitive responses are accompanied by changes in subjective psychological states. Methodological limitations within the primary studies, particularly inadequate reporting of allocation concealment, limited formal assessment of blinding effectiveness, and the general absence of preregistered protocols or prespecified analysis plans, reduce confidence in the available evidence. Additionally, the review protocol was registered retrospectively after completion of the review procedures. Although this registration provides a publicly accessible record, the absence of prospective registration limits the ability to verify that the review methods and analyses were prespecified. Given these limitations, the findings should be interpreted cautiously and should not be considered definitive evidence of cognitive or psychological benefits of Caf-MR in trained individuals.

### 4.7. Practical Implications

First, Caf-MR may be considered a non-ingestive caffeine strategy for specific situations rather than a broadly effective cognitive or psychological ergogenic aid. Its potential value appears to lie in selected cognitive outcomes rather than in consistent improvements across cognitive domains or psychological states.

Second, trained individuals and practitioners should not expect Caf-MR to uniformly improve reaction time, response accuracy, mood, motivation, or mental effort. Cognitive benefits were observed only in selected tasks, suggesting that any effect is likely to depend on the specific cognitive demand and testing context. Psychological responses were even less consistent, with only isolated evidence of increased felt arousal. Caf-MR may therefore have potential applications in selected tasks and experimental contexts rather than serving as a general method for enhancing mental performance. Third, Caf-MR may be considered when caffeine ingestion is undesirable or impractical, as it provides a non-ingestive approach to caffeine administration. However, current evidence is insufficient to recommend a specific caffeine concentration, rinse duration, rinse frequency, nutritional condition, or time of day for maximizing cognitive or psychological responses. Given the variability in responses, trained individuals and practitioners may consider evaluating Caf-MR during training before its use in competition.

Finally, within a broader sports nutrition context, Caf-MR may be considered an optional strategy that complements, rather than replaces, established caffeine and nutrition practices. Until more consistent evidence is available, its practical application should remain individualized, with consideration of task demands and the specific purpose for which it is being used.

## 5. Conclusions

Although some studies reported improvements in selected cognitive outcomes, the small number of included studies and heterogeneity in cognitive assessments limit confidence in these findings. Current evidence remains insufficient to establish consistent cognitive or psychological benefits of Caf-MR in trained individuals. Cognitive responses varied across tasks and experimental contexts, although the factors underlying these differences remain uncertain. Methodological heterogeneity and limited assessment of blinding further restrict confidence in the available evidence. Future research should prioritize formally assessing blinding effectiveness, systematically comparing Caf-MR protocols under standardized experimental conditions, and using comparable cognitive and psychological outcome measures to better clarify the potential effects of Caf-MR in trained individuals.

## Figures and Tables

**Table 3:** Detailed risk-of-bias assessment of the included studies using the Cochrane RoB 2 tool for crossover trials.

Study	D1	DS	D2	D3	D4	D5	Overall
**Amor et al. [36]**	Low	Low	Low	Low	Low	Some Concerns	Some Concerns
**Boat et al. [37]**	Some Concerns	Low	Low	Low	Low	Some Concerns	Some Concerns
**Clarke et al. [18]**	Some Concerns	Low	Low	Low	Low	Some Concerns	Some Concerns
**Karayigit et al. [32]**	Some Concerns	Low	Low	Low	Low	Some Concerns	Some Concerns
**Oliveira et al. [38]**	Some Concerns	Low	Low	Low	Low	Some Concerns	Some Concerns
**Pirmohammadi** **et al. [34]**	Some Concerns	Low	Low	Low	Low	Some Concerns	Some Concerns
**Şahin et al. [35]**	Some Concerns	Low	Low	Low	Low	Some Concerns	Some Concerns
**Tallis et al. [39]**	Some Concerns	Low	Low	Low	Low	Some Concerns	Some Concerns
**Virdinli et al. [33]**	Some Concerns	Low	Low	Low	Low	Some Concerns	Some Concerns
**Yüce et al. [40]**	Some Concerns	Low	Low	Low	Some Concerns	Some Concerns	Some Concerns

D1: Randomization process; DS: Risk of bias arising from period and carryover effects; D2: Deviations from intended interventions; D3: Missing outcome data; D4: Measurement of the outcome; D5: Selection of the reported result. Risk-of-bias judgments: Low, low risk of bias; Some concerns, some concerns regarding risk of bias.

**Table 4:** Detailed methodological quality assessment of the included studies using the PEDro scale.

Study	Item1	Item2	Item3	Item4	Item5	Item6	Item7	Item8	Item9	Item10	Item11	Item12	Total	Rating
Amor et al. [36]	Yes	1	1	1	0	0	0	1	1	1	1	0	7	Good
Boat et al. [37]	Yes	1	0	1	1	0	1	1	1	1	1	0	8	Good
Clarke et al. [18]	Yes	1	0	1	0	0	0	1	1	1	1	0	6	Fair
Karayigit et al. [32]	Yes	1	0	1	0	0	0	1	1	1	1	0	6	Fair
Pirmohammadi et al. [34]	Yes	1	0	1	0	0	0	1	1	1	1	0	6	Fair
Şahin et al. [35]	Yes	0	0	1	1	0	0	1	1	1	1	0	6	Fair
Tallis et al. [39]	Yes	1	0	1	1	0	1	1	0	1	1	0	7	Good
Virdinli et al. [33]	Yes	1	0	1	0	0	0	1	1	1	1	1	7	Good
Oliveira et al. [38]	Yes	1	0	1	0	0	0	1	1	1	1	0	6	Fair
Yüce et al. [40]	Yes	1	0	1	0	0	0	1	1	1	1	0	6	Fair

## Data Availability

No new primary data were collected in this study. All information analyzed in this systematic review was obtained from previously published studies cited in the article.

## Supplementary Materials

The following supporting information can be downloaded at https://www.mdpi.com/article/10.3390/nu18213695/s1; Table S1: Database-specific search strategies for cognitive and psychological outcomes related to caffeine mouth rinse. Supplementary File S1: PRISMA 2020 checklist.
