# Supplementary material for: Effects of Caffeine Mouth Rinse on Cognitive Performance and Psychological Responses in Trained Individuals: A Systematic Review
Source: Nutrients. 2026 Nov 7;18(21):3695. doi: 10.3390/nu18213695 (PMC13765250; doi:10.3390/nu18213695)
Supplement: Supplementary file 1 [file nutrients-18-03695-s001.zip › Table S1.pdf]

Supplementary Table S1. Database-specific search strategies for cognitive and psychological outcomes related to caffeine mouth rinse.

- *Search strategy for cognitive outcomes*

Database	Search strategy	Filter	Results	Date search
PubMed	(caffeine[Title/Abstract] OR caffeinated[Title/Abstract] OR coffee[Title/Abstract]) AND ("mouth rinse"[Title/Abstract] OR "mouth rinsing"[Title/Abstract] OR "oral rinse"[Title/Abstract] OR "oral rinsing"[Title/Abstract] OR mouthwash[Title/Abstract]) AND (cognit*[Title/Abstract] OR attention*[Title/Abstract] OR "reaction time"[Title/Abstract] OR vigilance[Title/Abstract] OR memory[Title/Abstract] OR "executive function"[Title/Abstract] OR "inhibitory control"[Title/Abstract] OR psychomotor[Title/Abstract])	N/A	15	2026/9/24
Scopus	TITLE-ABS-KEY(caffeine OR caffeinated OR coffee) AND TITLE-ABS-KEY("mouth rinse" OR "mouth rinsing" OR "oral rinse" OR "oral rinsing" OR mouthwash) AND TITLE-ABS-KEY(cognit* OR attention* OR "reaction time" OR vigilance OR memory OR "executive function" OR "inhibitory control" OR psychomotor)	Document type: Article; Language: English	15	2026/9/24
Web of Science	TS=(caffeine OR caffeinated OR coffee) AND TS=("mouth rinse" OR "mouth rinsing" OR "oral rinse" OR "oral rinsing"	Document type: Article	18	2026/9/24

	OR mouthwash) AND TS=(cognit* OR attention* OR "reaction time" OR vigilance OR memory OR "executive function" OR "inhibitory control" OR psychomotor)			
Embase	(caffeine OR caffeinated OR coffee):ti,ab,kw AND ("mouth rinse" OR "mouth rinsing" OR "oral rinse" OR "oral rinsing" OR mouthwash):ti,ab,kw AND (cognit* OR attention* OR "reaction time" OR vigilance OR memory OR "executive function" OR "inhibitory control" OR psychomotor):ti,ab,kw	N/A	21	2026/9/24
Science Direct	(caffeine OR coffee)AND("mouth rinse" OR "mouth rinsing" OR "oral rinse")AND(cognitive OR cognition OR "reaction time")	Document type: Article; Language: English	59	2026/9/24
Cochrane Library	#1 (caffeine OR caffeinated OR coffee) **[Title Abstract Keyword]** #2 ("mouth rinse" OR "mouth rinsing" OR "oral rinse" OR "oral rinsing" OR mouthwash) **[Title Abstract Keyword]** #3 (cognit* OR attention* OR "reaction time" OR vigilance OR memory OR "executive function" OR "inhibitory control" OR psychomotor) **[Title Abstract Keyword]** #4 #1 AND #2 AND #3	N/A	9	2026/9/24

Note: Owing to the Boolean operator and search-interface limitations of ScienceDirect, a simplified search strategy was used. The search terms were therefore more restricted than those used in the other databases, and potentially relevant records were further assessed during title/abstract and full-text screening.

- *Search strategy for psychological outcomes*

Database	Search strategy	Filter	Results	Date search
PubMed	(caffeine[Title/Abstract] OR caffeinated[Title/Abstract] OR coffee[Title/Abstract]) AND ("mouth rinse"[Title/Abstract] OR "mouth rinsing"[Title/Abstract] OR "oral rinse"[Title/Abstract] OR "oral rinsing"[Title/Abstract] OR mouthwash[Title/Abstract]) AND (psycho*[Title/Abstract] OR mood[Title/Abstract] OR affect*[Title/Abstract] OR arousal[Title/Abstract] OR motivation*[Title/Abstract] OR pleasure[Title/Abstract] OR "mental fatigue"[Title/Abstract] OR feeling*[Title/Abstract])	N/A	21	2026/9/24
Scopus	TITLE-ABS-KEY(caffeine OR caffeinated OR coffee) AND TITLE-ABS-KEY("mouth rinse" OR "mouth rinsing" OR "oral rinse" OR "oral rinsing" OR mouthwash) AND TITLE-ABS-KEY(psycho* OR mood OR affect* OR arousal OR motivation* OR pleasure OR "mental fatigue" OR feeling*)	Document type: Article; Language: English	36	2026/9/24
Web of Science	TS=(caffeine OR caffeinated OR coffee) AND TS=("mouth rinse" OR "mouth rinsing" OR "oral rinse" OR "oral rinsing" OR mouthwash) AND TS=(psycho* OR mood OR affect* OR arousal OR motivation* OR pleasure OR "mental fatigue" OR feeling*)	Document type: Article	38	2026/9/24
Embase	(caffeine OR caffeinated OR coffee):ti,ab,kw AND ("mouth rinse" OR "mouth rinsing" OR "oral rinse"	N/A	35	2026/9/24

	OR "oral rinsing" OR mouthwash):ti,ab,kw AND (psycho* OR mood OR affect* OR arousal OR motivation* OR pleasure OR "mental fatigue" OR feeling*):ti,ab,kw			
ScienceDirect	(caffeine OR coffee) AND ("mouth rinse" OR "mouth rinsing" OR "oral rinse") AND (psychological OR mood OR arousal)	Document type: Article; Language: English	77	2026/9/24
Cochrane Library	#1 (caffeine OR caffeinated OR coffee) [Title Abstract Keyword] #2 ("mouth rinse" OR "mouth rinsing" OR "oral rinse" OR "oral rinsing" OR mouthwash) [Title Abstract Keyword] #3 (psycho* OR mood OR affect* OR arousal OR motivation* OR pleasure OR "mental fatigue" OR feeling*) [Title Abstract Keyword]<br>#4 #1 AND #2 AND #3	N/A	22	2026/9/24

Note: Owing to the Boolean operator and search-interface limitations of ScienceDirect, a simplified search strategy was used. The search terms were therefore more restricted than those used in the other databases, and potentially relevant records were further assessed during title/abstract and full-text screening.
